# YAP/Yorkie in the germline modulates the age-related decline of germline stem cells and niche cells

**DOI:** 10.1371/journal.pone.0213327

**Published:** 2019-04-03

**Authors:** Deepthy Francis, Bhavna Chanana, Beatriz Fernandez, Benjamin Gordon, Tiffany Mak, Isabel M. Palacios

**Affiliations:** 1 University of Cambridge, Cambridge, United Kingdom; 2 School of Biological and Chemical Sciences, Queen Mary University of London, Mile End Road, London, United Kingdom; National Cancer Institute, UNITED STATES

## Abstract

The properties and behaviour of stem cells rely heavily on signaling from the local microenvironment. At the apical end of *Drosophila* testis, self-renewal and differentiation of germline stem cells (GSCs) are tightly controlled by distinct somatic cells that comprise a specialised stem cell niche known as the hub. The hub maintains GSC homeostasis through adhesion and cell signaling. The Salvador/Warts/Hippo (SWH) pathway, which suppresses the transcriptional co-activator YAP/Yki via a kinase cascade, is a known regulator of stem cell proliferation and differentiation. Here, we show that increasing YAP/Yki expression in the germline, as well as reducing Warts levels, blocks the decrease of GSC numbers observed in aging flies, with only a small increase on their proliferation. An increased expression of YAP/Yki in the germline or a reduction in Warts levels also stymies an age-related reduction in hub cell number, suggesting a bilateral relationship between GSCs and the hub. Conversely, RNAi-based knockdown of YAP/Yki in the germline leads to a significant drop in hub cell number, further suggesting the existence of such a SC-to-niche relationship. All together, our data implicate the SWH pathway in *Drosophila* GSC maintenance and raise questions about its role in stem cell homeostasis in aging organisms.

## Introduction

Studies from invertebrates to mammals demonstrate that stem cells (SCs) and their niches are crucial for maintaining tissue homeostasis. By mechano-chemical cues, stem cell niches regulate SC maintenance and survival. As shown for example in *Drosophila melanogaster* [1–5], the SCs that remain in contact with the niche receive these cues and self-renew, while those that lose contact with the niche differentiate. Despite this critical requirement for the niche in tissues, little is known about how its function is regulated.

*Drosophila* testes provide an excellent system to study SCs and their niches *in vivo* ([6], reviewed in [7]). The hub, formed by somatic cells, is a specialized niche essential for maintaining testes SC identity. Both the germline (GSCs) and the somatic cyst (CySC) SCs are adjacent to the hub (S1 Fig). The hub cells secrete the Jak-Stat ligand Unpaired (Upd) to promote SC maintenance [1, 2, 8]. The orientation of the SC spindle, perpendicular to the hub, allows for one daughter cell to lose contact with the hub, and differentiate (S1C Fig) [9].

In mammals, infertility in old males seems to result from the niche failing to support the balance between SC self-renewal and differentiation [10, 11]. In *Drosophila*, older hubs substantially decrease its production of the signal Upd, an event that correlates with decline in sperm production [12]. Aging also results in a modest loss of hub cell number [12, 13]. Experiments addressing the effect of age on GSCs resulted in ambiguous results, with one study supporting that the overall number of GSCs is maintained during aging [13], while others reporting a loss of GSCs in aging testes [12, 14]. Therefore, although the importance of the hub is well defined, more work is required to determine the mechanisms of GSC-hub homeostasis.

Mechano-chemical interactions may be the key to help maintain a healthy hub in order to allow SC maintenance and sperm production to carry on for as long as possible. SCs must respond to growth demands while limiting the potential for tumorigenesis. The SWH tumor-suppressor pathway modulates pluripotent lineage differentiation in mouse embryos [15, 16], as well as proliferation of intestinal and epidermal SCs across organisms [17–20]. Here we address the role of aging in hub and GSC number. In addition, we investigate the function of the SWH pathway in maintaining hub cell and SC numbers in aging testes.

## Results and discussion

### The number of GSCs declines with age

**Fig 1:**
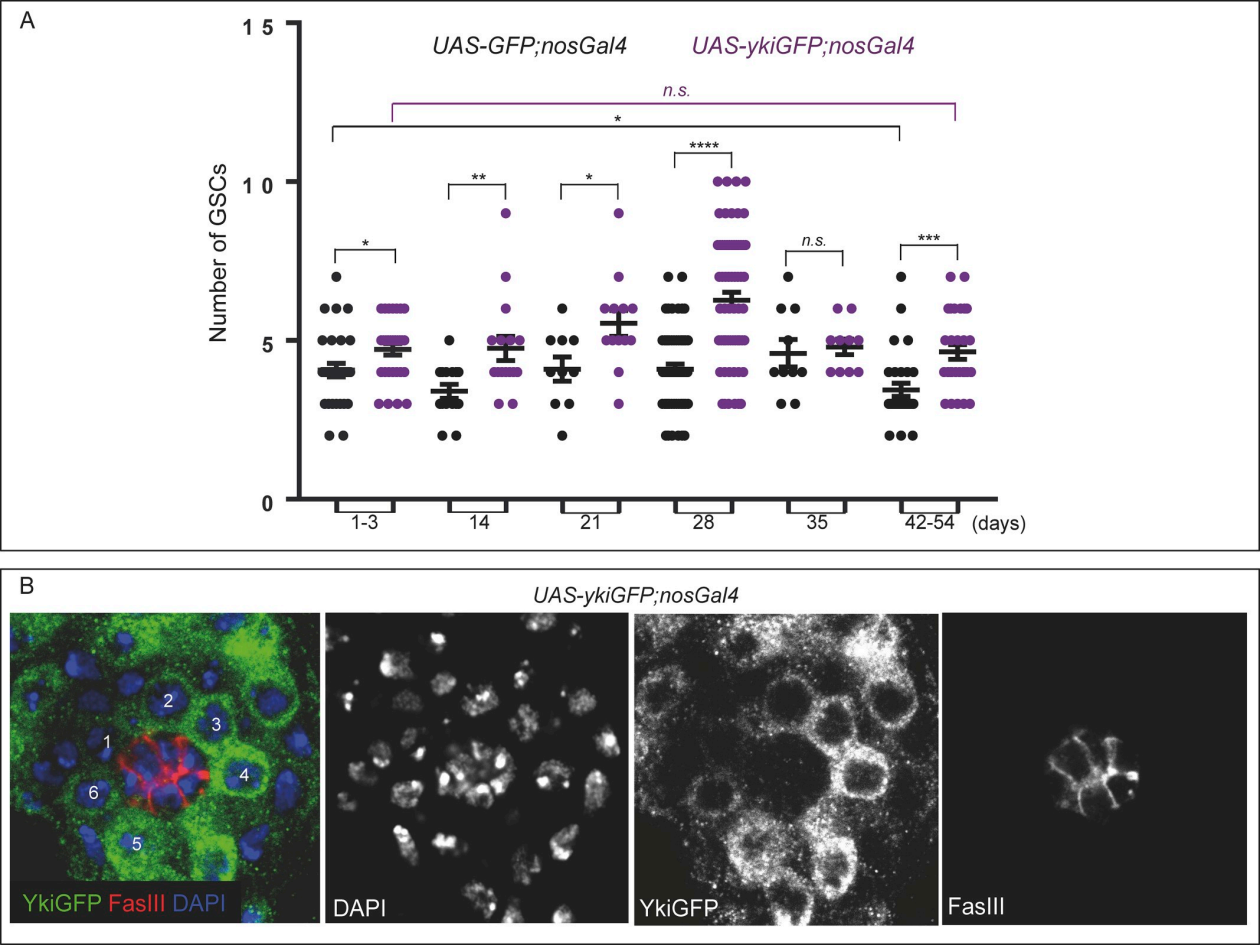
Higher levels of YAP/Yki in the germline increases the number of GSCs and blocks their age-related reduction. **A)** Plot with individual data points showing the number of GSCs in aging testes expressing GFP (black) or YAP/YkiGFP (purple) in the germline. This quantification illustrates that the average number of GSCs in GFP control testes drops with age: 1–3 (4.07±0.21, n = 30), 14 (3.40±0.21, n = 15), 21 (4.10±0.38, n = 10), 28 (4.10±0.16, n = 63), 35 (4.60±0.43, n = 10), and 42–54 days-old testes (3.45±0.21, n = 29). This reduction in number of GSCs is also observed in w^118^ testes, with 6.59±0.20 (n = 27) at 7 days, and 4±0.21 (n = 10) at 28 days (S2B Fig). We are depicting the p values as calculated only between 1 and 42–54 days. This quantification also illustrates that the average number of GSCs in testes expressing YAP/Yki in the germline does not seem to drop with age: 1–3 (4.72±0.19, n = 29), 14 (4.75±0.38, n = 16), 21 (5.54±0.40, n = 13), 28 (6.27±0.24, n = 67), 35 (4.80±0.25, n = 10) and 42–54 days-old testes (4.64±0.23, n = 28) (Unpaired student ‘t’ test: n.s. not statistically significant between 1 and 42–54 days). Furthermore, unpaired student ‘t’ test on the values obtained in YkiGFP;nosGal4 testes compared to controls also suggests that testes over-expressing YAP/YkiGFP in the germline show a significant increase in the number of GSCs when compared to controls (Unpaired student ‘t’ test *p<0.05, **p<0.01, ***p<0.001 and ****p<0.0001). (see raw data file for details) **B)** UAS-ykiGFP;nosGal4 testes express YkiGFP in all germline cells, including GSCs. The hub (FasIII) and DAPI are red and blue in merge panels, respectively. This representative image is a single plane, but the quantification was done in sections that cover the whole region and all cells (see S1 Movie and Methods for further details).

To study whether the number of GSCs declines with age, we quantified the number of GSCs in 1–54 days-old males by driving expression of UAS-GFP with the germline driver nanos-Gal4-VP16 (nosGal4 from here on). This quantification showed that the number of GSCs drops with age (Fig 1, Table 1 and S2B Fig). In UAS-GFP;nosGal4 testes, the absolute number of GSCs is lower at 42–54 days than at 1–3 days. This age-related reduction in GSC number is also observed in 7–28 days wild-type (w^1118^) and UAS-GFP males, where GSCs were detected by their position next to the niche, and being positive for the germline marker Vasa (S2B Fig, Table 1). Other possible germline drivers, such as vasa-Gal4 and hsp83-Gal4 did not drive expression in the GSCs in our hands (S2A Fig) [21–24], and limited our study to using the nosGal4 driver when wanting to modulate levels in the GSCs. Our quantification in various aging testes confirms that the number of GSCs drops with age, and in a similar manner to comparable genetic backgrounds previously analysed (Table 1) [12, 13].

**Table 1:** This table summarises our data and the data from Boyle et al., and Wallenfang et al., publications that previously looked at aging testes.

Experiment	Genotype	Cell	1–3 Days	7 Days	14 Days	21 Days	28 Days	35 Days	42–54 Days
***Boyle et al*., *Cell Stem Cell 2007*** *(Error as SEM)*	OregonR (Error as SEM)	GSC	8.27±0.23				5.85±0.25 (30 days)		5.1±0.23 (50 days)
		Hub	11.4±0.48						9.3±0.35 (50 days)
***Wallenfang et al*., *Aging Cell 2006*** *(Error as SD)*	w^1118^ (Error as SD)	GSC	5.6±1.1 (0 day)				4.2±1.3 (35 days)		
		Hub	9.6±2.6 (0 day)				6.4±2.3 (35 days)		
***WT vs*. *GFP*** *(Error as SEM)*	**w**^**1118**^	**GSC**		6.59±0.20	5.0±0.29		4.0±0.21		
	**UAS-GFP**	**GSC**		6.25±0.41	5.5±0.18		4.7±0.21		
***Yorkie Overexpression*** *(Error as SEM)*	UAS-GFP;nosGal4	GSC	4.07±0.21		3.40±0.21	4.10±0.38	4.10±0.16	4.60±0.43	3.45±0.21
	UAS-ykiGFP;nosGal4	GSC	4.72±0.19		4.75±0.38	5.54±0.40	6.27±0.24	4.80±0.25	4.64±0.23
***Yorkie Overexpression*** *(Error as SEM)*	**UAS-GFP;nosGal4**	**Hub**	6.28±0.21		6.26±0.22				
	**UAS-ykiGFP;nosGal4**	**Hub**	6.60±0.25		7.66±0.26				
***Yorkie Overexpression and Knock-down*** *(Error as SEM)*	UAS-GFP;nosGal4	Hub		6.20±0.31 (n.s)		5.90±0.41 (n.s)	6.72±0.18 (p = 0.0037)	5.60±0.34 (n.s.)	5.17±0.22 (n.s.)
	UAS-ykiRNAi;nosGal4	Hub		5.71±0.27		6.00±0.49	5.67±0.29	6.10±0.23	5.30±0.26
	UAS-ykiGFP;nosGal4	Hub		6.67±0.32 (p = 0.0272)		7.85±0.71 (p = 0.0433)	8.30±0.19 (p<0.0001)	6.90±0.43 (n.s.)	7.18±0.37 (p = 0.0002)
***EdU*** ***% of testes***^***1***^ *(Error as SEM)*	**UAS-GFP;nosGal4**	**GSC**			50%^1^		28%^1^		10%^1^ (53 days)
	**UAS-ykiGFP;nosGal4**	**GSC**			79%^1^		54%^1^		40%^1^ (53 days)
***EdU*** ***% of GSCs***^***2***^ *(Error as SEM)*	**UAS-GFP;nosGal4**	**GSC**			49.04±9.8%^2^		20±7.21%^2^		5.0±5%^2^ (53 days)
	**UAS-ykiGFP;nosGal4**	**GSC**			73.89±7.6%^2^		32.03±7%^2^		23.36±9.55%^2^ (53 days)
	UAS-GFP; wts/nosGal4	Hub	8.57±0.33	6.16±0.21			8.67±0.26		
***warts***^***x1***^ ***heterozygous*** *(Error as SEM)*	UAS-ykiGFP; wts/nosGal4	Hub	10.50±0.52	9.29±0.27			9.35±0.30		
	UAS-GFP; wts/nosGal4	GSC	6.50±0.44	4.48±0.31			4.83±0.26		
	UAS-ykiGFP; wts/nosGal4	GSC	7.90±0.43	6.43±0.37			5.82±0.33		

Note that the published work quantified both wild-type flies (oregonR, ‘real wild-type’), and *w*^*118*^, a mutant that is often used as control, as many *Drosophila* strains are with this mutation in the background. We mainly quantified nanosGal4;UASGFP flies, which may explain the differences observed at 1–3 days between the published and our work. We are only able to directly compare our quantification in *w*^*118*^ with published data in the 28 days column, where we can see that our values (3–5 cells) are similar to the other 2 values. Also note that one paper used SEM while the other used SD for the stats. When comparing the wild-type flies from the published work, to our nanosGal4;UASGFP flies, you can see that the differences are not major, although nanosGal4;UASGFP numbers are slightly lower. This is also the case when comparing nanosGal4;UASGFP flies to *w*^*118*^ flies in our hands. The nanosGal4;UASGFP flies are the right control in our study. In our cell number quantifications, average data are presented as mean ± SEM.

^1^% of testes with any EdU positive GSC.

^2^% of GSCs positive for EdU

### Higher YAP/Yki levels in the germline increase the number of GSCs

Mutations in the core components of the SWH pathway, such as the kinase Warts (Wts), can often be mimicked by over-expression of the transcriptional co-activator Yorkie (Yki, YAP in mammals). In order to examine whether the SWH pathway may contribute to the age-related variation of GSC number, we over-expressed YAP/YkiGFP in the germline using nosGal4 and quantified the total number of GSCs (Fig 1). Testes over-expressing YAP/YkiGFP in the germline showed a significant increase in the number of GSCs when compared to controls. This increase is initially detected as a trend at 1–3 days (4.72±0.19, n = 29, controls 4.07±0.21, n = 30), but becomes more prominent as the testes age, with 4.75±0.38 (n = 16), 5.54±0.40 (n = 13), 6.27±0.24 (n = 67), 4.80±0.25 (n = 10) and 4.64±0.23 (n = 28) GSCs in 14, 21, 28, 35 and 42–54 days YAP/YkiGFP testes, respectively, compared to 3.40±0.21 (n = 15), 4.10±0.38 (n = 10), 4.10±0.16 (n = 63), 4.60±0.43 (n = 10) and 3.45±0.21 (n = 29) GSCs in 14, 21, 28, 35 and 42–54 days control testes, respectively.

Since we observed an increase in GSC number when YAP/Yki was over-expressed in the germline, we then analysed whether mutations in wts had a similar effect. As null mutations of wts are homozygous lethal [25], we analysed the effect of heterozygous mutations. Males with one copy of a null wts mutation alone (wts/+) displayed a significant increase in the number of GSCs, from 4.07 (±0.21, n = 30) and 4.10 (±0.16, n = 63) in GFP controls to 6.50 (±0.44, n = 14, p<0.0001) and 4.83 (±0.26, n = 18, p = 0.0209) in GFP; wts/+ (UAS-GFP; FRT82B wts/nosGal4), at 1–3 days and 28 days testes, respectively (Table 1). However, wts/+ had only a combinatorial effect on GSC number when YAP/Yki was over-expressed in the germline at 1–3 days, from 4.72±0.19 (n = 29) in Yki testes to 7.90±0.43 (n = 10, p<0.0001) in Yki; wts/+ (UASYkiGFP; FRT82B wts/nosGal4, S2C Fig, Table 1).

### Increased YAP/Yki levels in the germline prevent the age-related reduction of GSCs

The quantification of GSC number also revealed that, contrary to controls, the average number of GSCs did not drop significantly over the course of aging in YAP/YkiGFP;nosGal4 testes (Fig 1). Contrary to the over-expression of YAP/YkiGFP in the germline, the number of GSCs still drops with age in testes over-expressing YAP/Yki in the hub cells (by the driver upd-Gal4, S3 Fig), or in the somatic cells (by the driver Trafficjam-(Tj)-Gal4, S4 Fig). All these findings together suggest that the SWH pathway could be acting in the GSCs, to reduce an age-dependent drop in their number. Our findings correlate well with recent findings in the developing female germline, where reducing or increasing Yki results in a lower and a higher number of larval germ cells, respectively [26].

There are various mechanisms by which the SWH pathway might regulate GSC number in aging testes. One possibility is that the SWH pathway could reduce GSC loss through increasing physical adhesions, for example by maintaining high E-cadherin levels at the membrane, which has been shown to decrease with age [12, 27, 28]. We investigated this possibility and observed that in control testes the levels of E-cadherin are lower at 45 days than at 21 days, while the levels of E-cadherin barely drop in 45 days YAP/Yki over-expressing testes, compared to 21 days (representative image in S5A Fig, n = 5 for each sample).

### Impact of YAP/Yki over-expression on the proportion of testes with EdU positive GSCs

The SWH pathway might also maintain GSCs in aging testes by regulating gonialblasts de-differentiation to a GSC [29], or by modulating the number of GSC spindles that are parallel—instead of perpendicular—to the hub, resulting in two daughter SCs upon division. Finally, the SWH pathway may also maintain GSCs in aging testes by modulating GSC proliferation. Here we investigated whether changes in cell proliferation may contribute to the decline in GSCs with age, and the effects of the SWH pathway. We addressed this possibility by using the marker of cell replication EdU in testes where the GSCs are marked with GFP, which allowed us to identify EdU positive GSCs.

**Fig 2:**
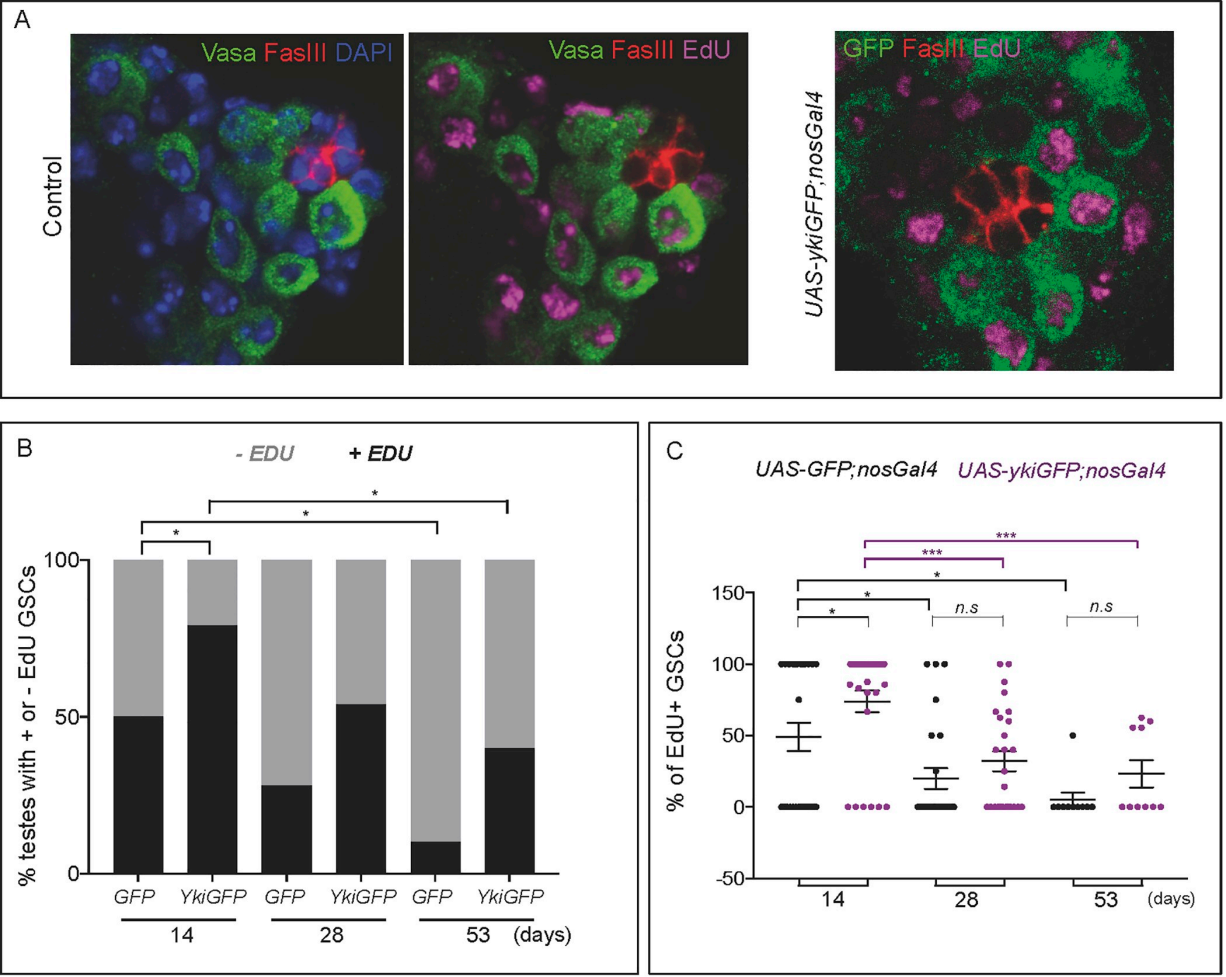
Impact of YAP/Yki over-expression on the incorporation of EdU in GSCs. **A)** Representative images of EdU labelling in controls (w^118^) or testes that over-express YAP/Yki in the germline. DAPI (DNA) in blue, EdU in magenta, and FasIII in red. Left panels show Vasa in green, while right panel shows YkiGFP in green. **B)** Proportion of testes with any EdU+ GSC (black) in UAS-GFP;nosGal4 and UAS-ykiGFP;nosGal4 flies. This quantification shows that the proportion of control testes with any EdU+ GSC drops significantly with age: in males expressing GFP, 50% (n = 26) at 14 days to 10% (n = 10) at 53 days (the chi-square statistic is 4.8623, p = 0.27449), while in males expressing YAP/Yki, 79% (n = 28) at 14 days to 40% (n = 10) at 53 days (the chi-square statistic is 5.0736, p = 0.024293). Our data also shows that over-expression of YAP/Yki in the germline increases the proportion of testes with any EdU+ GSCs when compared to GFP controls at 14 day-old males (the chi-square statistic is 4.8258, p = 0.028037), and with increasing trends at 28–53 days as well (28 days, the chi-square statistic is 4.8258, p = 0.060813; 53 days, the chi-square statistic is 4.8258, p = 0.121335). *p<0.05. **C)** Percentage of GSCs labelled with EdU in testes that express GFP (black) or YAP/YkiGFP (purple) in the germline. The percentage of EdU-labelled GSCs decreases with age in both phenotypes (YAP/Yki: 73.89±7.6%, n = 28; 32.03±7%, n = 26 and 23.36±9.55%, n = 10 in 14, 28 and 53 days, respectively; GFP 49.04±9.85%, n = 26, 20±7.21%, n = 25 and 5±5%, n = 10 in 14, 28 and 53 days, respectively). *p<0.05, ***p<0.001. (See S1 Dataset, raw data file for details) All images here are a single plane, but the quantification was done in sections that cover the whole region and all cells (see S1 Movie and Methods for further details).

We quantified the proportion of testes with any EdU+ GSCs in flies of increasing age (Fig 2B). Our results show that the proportion of control testes with EdU+ GSCs drops significantly from 14 to 53 days (50%, n = 26 and 10%, n = 10) (p = 0.0274). This age-related reduction in the percentage of testes with EdU+ GSCs is also observed in males expressing YAP/Yki in the germline, with a drop from 79% to 40% from 14 (n = 28) to 53 days (n = 10) (p = 0.0243). Our data also shows that over-expression of YAP/Yki in the germline increases the proportion of testes with any EdU+ GSCs when compared to GFP controls, with a 29% increase for 14 day-old males (Fig 2B) (p = 0.028), and with increasing trends at 28–53 days as well. Thus, the proportion of testes with EdU+ GSCs drops with age in both controls and YAP/Yki testes. In addition, the proportion of testes with EdU+ GSCs is higher when YAP/Yki is over-expressed in the germline than in controls.

### Impact of YAP/Yki over-expression on the proportion of EdU positive GSCs

We then quantified the percentage of GSCs labelled with EdU in control and YAP/Yki testis, and this analysis showed that the number of EdU positive GSCs in each testis also decreases with age. For the YAP/Yki testes, we observed a stark 42% decrease of the mean percentage of EdU+ GSCs from 14 to 28 days testes, and a 9% decrease from 28 to 53 days. For control GFP testes, we observed a 29% decrease of the mean percentage of EdU+ GSCs from 14 to 28 days testes, and a 15% decrease from 28 to 53 days (Fig 2C). These values show a clear loss of EdU+ GSCs in 53 days testes compared to 28 days testes, and 28 days testes compared to 14 days testes. This finding could suggest that GSC proliferation decreases with age in both control testes and in testes over-expressing YAP/Yki in the germline.

Contrary to the proportion of testes with any EdU+ GSCs, where we observe that it is always higher when YAP/Yki is over-expressed in the germline than in controls, YAP/Yki over-expression did not result in a strong increase in the number of EdU+ GSCs in each testis, although there seems to be a significant slight increase at 14 days (Fig 2C). This supports the findings that the SWH pathway is not required for proliferation control in the adult germline [30, 31]. When quantifying the proportion of EdU+ GSCs only in testes that had at least one EdU+ GSC, we observed the same trend.

Our results suggest that GSC proliferation may decrease with age, which confirms previous findings [13]. This reduction in proliferation probably contributes to the age-related reduction in GSC number observed in controls (UAS-GFP;nosGal4 in Fig 1, and UAS-GFP and w^118^ in S2 Fig). However, in males that express YAP/Yki in the germline the average number of GSCs is maintained with age (Fig 1), although the proportion of EdU+ GSCs drops with age in these testes (Fig 2C). This suggests that Yki may not only function through regulating proliferation, but that other mechanisms, which might be independent of proliferation, may also exist in order to protect the GSC pool from age-associated loss. One of these mechanisms is probably higher adhesion of GSCs to the hub. Whether the slightly higher percentage of EdU+ GSCs in the YAP/Yki over-expressing 14 days testes (Fig 2C) also contributes to the average number of GSCs being maintained with age in these testes needs further investigation.

### SWH acts in the germline to regulate hub cell number

**Fig 3:**
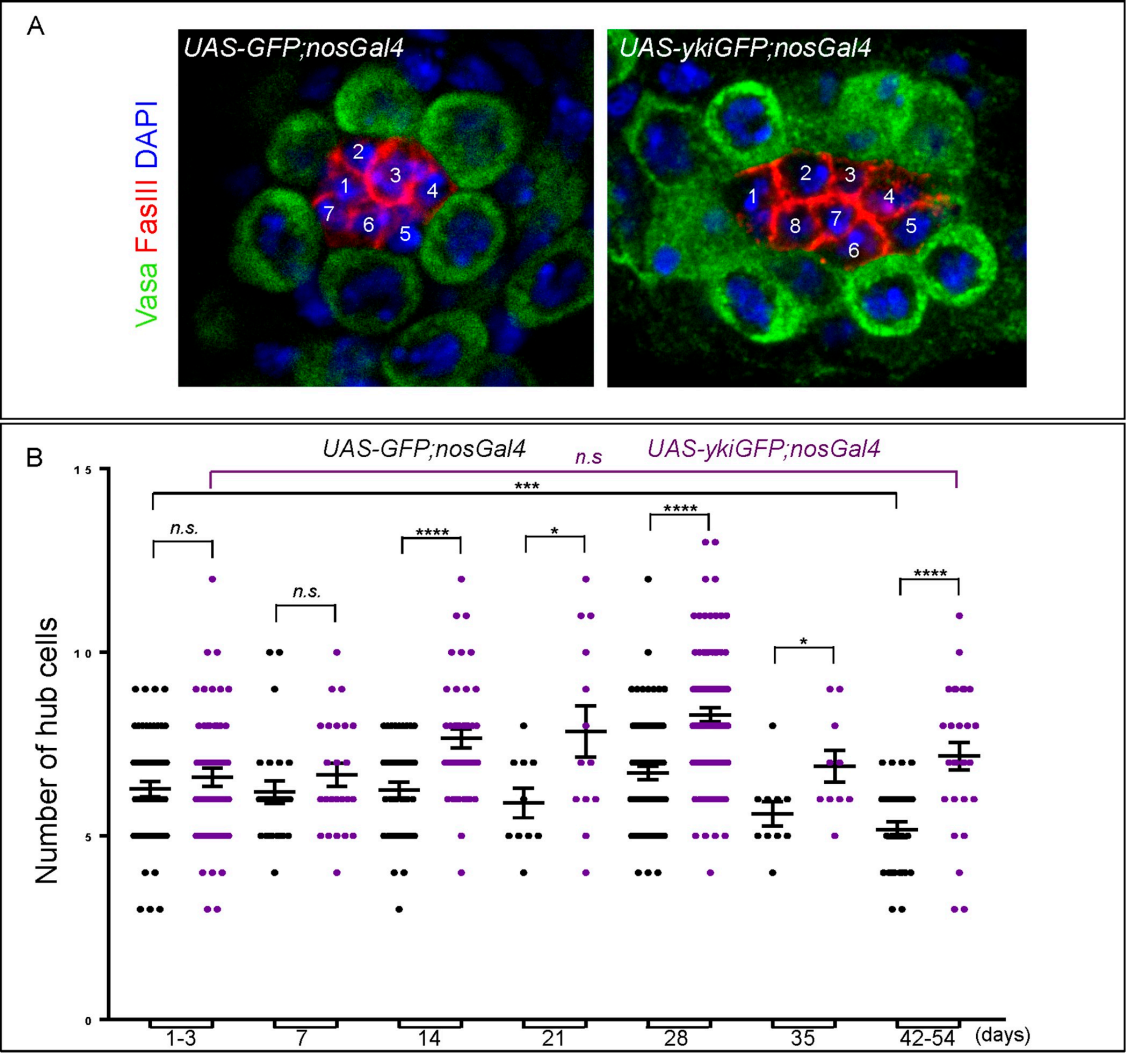
Higher levels of YAP/Yki in the germline induce an increase in the number of hub cells. **A)** DAPI in blue, FasIII in red, Vasa in green and GFP not shown. White digits indicate position of single hub cells. **B)** Plot with individual data points showing the number of hub cells in UAS-GFP;nosGal4 (black) and UAS-ykiGFP;nosGal4 (purple) testes. The average numbers are: *GFP*: 1 day 6.66±0.47 (n = 15), 14 days 6.29±0.22 (n = 38), 28 days 6.96±0.19 (n = 60), and 48–51 days 5.77±0.36 (n = 10). *YAP/Yki*: 1 day 7.26±0.58 (n = 15), 14 days 7.62±0.26 (n = 40), 28 days 8.31±0.25 (n = 60), and 48–51 days 8.54±0.41 (n = 11). (See S1 Dataset, raw data file for details). Unpaired student ‘t’ test on the values obtained in YkiGFP;nosGal4 testes compared to controls suggests that testes over-expressing YAP/YkiGFP in the germline show a significant increase in the number of hub cells when compared to controls. This increase becomes more prominent in testes that are older than 7 days (*p<0.05, ***p<0.001 and ****p<0.0001).

Signals coming from the niche regulate SC biology, but it is also possible that signals from SCs impact on niche function, as previously suggested in *Drosophila* ovaries [32]. Since higher levels of YAP/Yki in the germline result in more GSCs, we next quantified the total number of hub cells in these testes. As with GSCs, the average number of hub cells in testes over-expressing YAP/Yki in the germline is higher than in controls (Fig 3, Table 1). Higher number of hub cells when YAP/Yki is over-expressed in the germline is observed in 14, 21, 28, 35 and 42–54 days-old testes, with a strong trend for more hub cells when YAP/Yki is over-expressed in 1–3, and 7 days-old germline.

We observed that the number of hub cells has a tendency to increase with age in testes that expressed YAP/Yki in the germline, (6.60±0.25, n = 53, and 7.18±0.37, n = 28, in 1–3 and 42–54 days-old, respectively, although statistical n.s.) while hub cell numbers tend to decline in aging controls (6.28±0.2, n = 54, and 5.17±0.22, n = 28, in 1–3 and 42–54 days-old, respectively, p = 0.0006), which is consistent with previous findings (Fig 3, Table 1) [12, 13]. Here, however, we show that this age related tendency to reduced hub cell numbers seems to be blocked by over-expressing YAP/Yki in the germline (Fig 3). Higher YAP/Yki in the germline did not induce the hub cells to enter mitosis, as they were never labelled with ethynyl deoxyuridine (EdU, Fig 2), or a phospho-histone-3 (PH3) antibody (S5B Fig). Contrary to the YAP/YkiGFP;nosGal4 testes, the over-expression of YAP/Yki in the hub cells (S3 Fig) or in the somatic cells (S4 Fig) did not result in an increase of the number of hub cells.

We also detected an increase of hub cell numbers in wts/+ testes, with 8.57±0.33 (n = 14), 6.16±0.21 (n = 19) and 8.67±0.26 (n = 18), compared to 6.28±0.21 (n = 54, p<0.0001), 6.20±0.31 (n = 25, n.s.) and 6.72±0.18 (n = 74, p<0.0001) control GFP testes, at 1–3, 7 and 28 days, respectively (Table 1). Similarly, YAP/Yki; wts/+ also showed an increased number of hub cells compared to YAP/Yki, with 10.50±0.52 (n = 10), 9.29±0.27 (n = 14) and 9.35±0.30 (n = 17) in YAP/Yki; wts/+, compared to 6.60±0.25 (n = 53, p<0.0001), 6.67±0.32 (n = 24, p<0.0001) and 8.30±0.19 (n = 92, p = 0.0057) at 1–3, 7 and 28 days, respectively.

### Knocking down YAP/Yki in the germline reduces the average number of hub cells

To further test the hypothesis that the SWH pathway might be acting in the germline to regulate hub cell number, we quantified the number of hub cells in testes with YAP/Yki knocked down in the germline by RNAi (Table 1). At 28 days, we observed a significant reduction in the average number of hub cells in testes expressing *yki* RNAi in the germline (5.67±0.29, n = 21), compared to testes expressing GFP (6.72±0.18, n = 74, p = 0.0037), or YAP/YkiGFP (8.30±0.19, n = 92, p<0.0001) in the germline. At 7 days, the average number of hub cells in testes expressing *yki* RNAi in the germline (5.71±0.27, n = 14) was lower than in testes over-expressing YAP/Yki in the germline (6.67±0.32, n = 24, p = 0.0272) and tended to be lower than in control testes (6.20±0.31, n = 25, p = 0.2382, Table 1). At 21, 35 and 42–54 days, the average number of hub cells in testes expressing *yki* RNAi in the germline was lower than in testes over-expressing YAP/Yki in the germline, but similar to control testes (Table 1). Thus, reducing the levels of endogenous YAP/Yki in the germline tends to result in a decrease in the number of hub cells, further suggesting a requirement for the SWH pathway in the germline to regulate the hub cell number in aging testes. We did not observe a reduction in the number of GSCs in the same *yki* RNAi testes, suggesting that the volume of the hub could still be large enough for maintaining the same number of GSCs, as previous shown [33]. It is also possible that the RNAi approach only results in a small reduction in the levels of YAP/Yki in these conditions, which is supported by the fact that at 21, 35 and 42–54 days, the average number of hub cells in testes expressing *yki* RNAi in the germline was similar to control testes.

All these findings together suggest that the SWH pathway acts in the GSCs to maintain their number in aging testes. Furthermore, it suggests the existence of a possible mechano-chemical signal from the GSCs to the hub in aging adults to maintain the number of hub cells. This suggestion correlates well with the finding that the SWH pathway regulates the secretion of ligands for other signaling pathways both in *Drosophila* intestine and in human mammary epithelial cells [34–37]. Our attempts to analyse the levels of YAP/Yki in aging GSCs by immunostaining with available antibodies have unfortunately not been successful, as we were unable to detect a specific signal for the YAP/Yki protein. Thus, the activity of the SWH pathway in aging germline cells needs further investigation.

## Concluding remarks

The importance of SCs for hub homeostasis described here adds to the observation that Shriveled, secreted by somatic cells and GSCs, contributes to hub maintenance during aging [38]. Our findings also correlate with a reversible plasticity of the female niche modulated by diet and age [39]. As for the male niche, invasive cells, presumably somatic CySCs, seem to augment the non-dividing population of niche cells [27], a possible mechanism for the higher number of niche cells we observe in our studies when YAP/Yki is over-expressed in the GSCs. However, the possibility of GSCs also entering the niche and contributing to the hub cell population, although never reported, has yet to be ruled out. Indeed, the GSC spindle lays perpendicular to the niche, which presumably applies a physical exertion on the hub cells. We could speculate that, in principal, this force could result in the incorporation of a GSC into the hub area, and in the conversion of this SC to a somatic fate.

The extent and mechanism of the influence of YAP/Yki in GSC and hub cell numbers remain to be fully elucidated, although a combination of higher adhesion by E-Cadherin, higher proliferation and CySCs-to-hub fate change may contribute. It is already known that YAP/Yki activity in CySCs results in a skew of the neutral competition [40]. In addition to protecting the GSC pool from age-associated loss, over-expression of YAP/Yki in the germline had a significant effect on increasing the hub cell number, suggesting a bilateral relationship between GSCs and the hub. This crosstalk may possibly be a core component of long-term GSC maintenance.

## Material and methods

### Fly Stocks/genetics

Stocks obtained from the Bloomington Drosophila Stock Center (NIH P40OD018537) or the Drosophila Genomics Resource Center (NIH2P40OD010949-10A1) are named BL or DGRC, respectively, followed by the stock number.

- *P{w[+mC] = GAL4*::*VP16-nos*.*UTR}CG6325[MVD1]* BL4937. This construct contains about 700bp of nos promoter, the nos 5′UTR, the FLAG epitope tag, Gal4-VP16, nos 3’UTRs and about 500bp of genomic DNA 3’ of the nos transcription unit. Expression of this driver mirrors expression of the endogenous gene nos in both the male and female germline, and it was used to perform rescue experiments of the developmental defects in *pex2* mutant spermatocytes [22].
- *w; P{UAS-GFP*.*nls}/CyO*; *MKRS/TM6B* (created from *w; P{UAS-GFP*.*nls}* BL4775).
- *w*;*P{w[+mC] = UAS-yki*.*GFP}4-12-1/CyO*; *MKRS/TM6B (created from yw*; *P{w[+mC] = UAS-yki*.*GFP} 4-12-1* BL28815).
- *w; UAS-yki-RNAi/CyO and w; UAS-yki* (gift from Dr N. Tapon)
- *y w; Sp/CyO;FRT82B warts*^*x1*^*/TM6B[41]*
- *w; E132-Gal4 (upd-Gal4*, gift from Dr M. Zeidler)
- *c587-Gal4* (gift from Dr F Karam Teixeira)
- *P{Hsp83-Gal4} and vasa-Gal4*: DGRC109996: w[*]; P{w[+mC] = vas-GAL4.*2*.*6}2 / CyO; MKRS / TM6B* or DGRC109996: y[*] w[*]; wg[Sp-1] / CyO; P{w[+mC] = vas-GAL4.*2*.*6}3 / TM6B*
- *TjG4-P{w[+mW*.*hs] = GawB}* DGRC104055.

Males with GFPnls or YAP/YkiGFP expression in germline cells were generated by crossing UAS-GFPnls or UAS-ykiGFP females with nosGal4 males. Males with GFPnls or YAP/YkiGFP expression in somatic cells were generated by crossing UAS-GFPnls or UAS-ykiGFP females with tjGal4/CyO males. To circumvent developmental phenotypes until eclosion due to YAP/Yki over-expression, these crosses were stored at 18°C from the day they were set, in order to minimise Gal4 expression. When the flies started to eclose, the collected male progeny were aged at 20–22°C, as high activation at 25°C severely affected the tissue architecture of the testes making any analysis difficult. To allow a comparison of results obtained across different genotypes and ages, we followed this protocol for all crosses, except where indicated. All testes were dissected at 1–54 days as indicated, where day 1 was considered a day post-eclosion.

### Quantification of the number of cells

The number of GSCs and niche cells were quantified in fixed samples by following DAPI for the nuclei, GFP or the germline marker Vasa for GSCs and the niche specific marker FasIII for niche cells. Images of planes at various depths representing all GSCs and niche cells were obtained using the Z-stack function of the microscope (see S1 Movie, representative movie of the approach to counting cells). GSCs were only counted if they were directly adjacent to the hub structure. In all quantifications, the error bar represents SEM in each case and average data are presented as mean±SEM. Unpaired student ‘t’ test: *p<0.05, **p<0.01, ***p<0.001 and ****p<0.0001.

### EdU labelling

EdU was used instead of the commonly used BrdU because EdU detection employs the ‘click’ chemistry reaction and does not involve heat or acid treatment that is required for BrdU. The EdU protocol was initially tested with control flies, where we detected a reliable proportion of testes with EdU labelled GSCs (Fig 2). The protocol also allowed for the identification of germline and hub cells by antibody staining against their specific markers, and showed that this *in vivo* labelling method is a reliable approach to mark SCs that have undergone mitosis. In particular, GSCs were clearly marked in testes expressing GFP or YAP/YkiGFP under the nosGal4 driver. Hub cells were never labelled with EdU under our conditions.

EdU was introduced into the flies through feeding, according to a BrdU labelling protocol adapted from [13]. Flies were first aged to the required age (e.g., 1, 7 days and so on), and then fed fresh yeast paste containing 10μM EdU every 24 hours for 2 days. The testes were then extracted from the animal, fixed and stained accordingly. Testes with EdU labelling were extracted in PBT 0.2% Tween-20 (PBS + 0.2% Tween-20) and fixed for 20 minutes in 4% paraformaldehyde (PFA). After 2x 10 minutes washes they were kept in methanol until further treatments. Before staining the methanol samples were washed 3x for 10minutes in PBT 0.2%. Then the testes were treated with 0.5% Triton-X 100 in PBS for 20 minutes. Two 10 minutes washes with 1x PBS were performed before incubating in the Click-iT reaction cocktail for 30 minutes, protected from light. The cocktail was then removed, washed once with PBS, and then blocked in PBT-10 (PBT + 10% BSA) for 1 hour. The rest of the protocol followed from the primary antibody incubation step as described below in Immunohistochemistry.

In all quantifications, the error bar represents SEM in each case and average data are presented as mean±SEM. Unpaired student ‘t’ test: *p<0.05, **p<0.01, ***p<0.001 and ****p<0.0001.

### Immunohistochemistry

All steps were performed at room temperature, unless described otherwise. All testes were dissected in PBT 0.2% Tween-20 and fixed for 20 minutes in 4% PFA. After washing twice with PBT, the testes were stored in methanol at -20°C. The testes were washed once with PBS, twice with PBT for 2x10 minutes, and “blocked” in PBT-10 for 1hr, before being incubated with primary antibody in PBT-1 (PBS + 1% BSA + 1% Tween-20) overnight with agitation at 4°C. After washing the testes 2x10 minutes with PBT-1, they were incubated with a 1:200 dilution of secondary antibody in PBT-1 for 2 hours. After 3x10 minutes washes in PBS, the samples were mounted in DAPI-Vectashield (Vector).

Antibodies: rabbit Vasa (1:5000, a gift from Prof. Paul Lasko), guinea pig Tj (1:10000, a gift from Dorothea Godt), chicken GFP (1:2000, Abcam), mouse FasIII (7G10) (1:100, DSHB), rabbit PH3 (1:500, Millipore). Secondary antibodies were Goat Anti-Chicken (DyLight 488, Abcam 96951), AlexaFluor568 goat anti-mouse (Molecular Probes A11004), AlexaFluor647 goat anti-rabbit (Molecular Probes A21245), and goat anti-guinea pig Cy3 (Novus Biologicals NB710-4179). DAPI in vectashield (Vector H-1200), was used for the visualization of DNA. All samples were examined under a 40x/1.3 or 63x/1.4–0.6 Oil DIC Plan-Neofluar objective, using a Leica LSM SP5 upright confocal microscope.

## Supporting information

S1 Fig UAS-GFP;nosGal4 testes express GFP in all germline cells, including GSCs. **A)** Left: scheme of testis. Middle: testis expressing UAS-GFP by the nosGal4 driver. Right: Apical tip: FasIII for hub cells (cyan), Vasa for germline (green), Traffic-jam for somatic lineage (Tj, in magenta), and DNA with DAPI (blue). The Vasa and Tj positive cells in contact with the hub are the GSCs and CySC, respectively.
**B)** UAS-GFP;nosGal4 testes express GFP in all germline cells, including GSCs. The hub is positive for FasIII (red in merge), and the somatic cells (CySC and somatic cyst) for Tj (blue in merge).
**C)** Alpha-tubulin-GFP reveals the orientation of the mitotic spindle in GSCs (arrow).
(TIFF)

S2 Fig **A)** Testes expressing GFP by the vasa-Gal4 driver stock DGRC109996 (left), YkiGFP by the vasa-Gal4 driver stock DGRC109997, and GFP by the hsp83-Gal4 driver (right). None of these Gal4 drivers induces expression in the GSCs.
**B) w^118^ and UAS-GFP testes show a decrease in the number of GSCs with age.** Plot with individual data points showing that the average number of GSCs. This quantification illustrates that in w^118^ flies, the number of GSCs in 7-days-old testes is 6.59±0.20 (n = 27), with a significant decrease at 14 (5±0.29, n = 10) and 28 days (4±0.21,n = 10). In UAS-GFP flies, the average number of GSCs in 7-days-old testes is 6.25±0.41 (n = 8), with a significant decrease at 14 (5.5±0.18, n = 8) and 28 days (4.7±0.21,n = 10). This age-related reduction in GSC number in w^1118^ and UAS-GFP 7–28 days-old males was observed by identifying GSCs by their position next to the niche, and by being positive for the germline marker Vasa. *p<0.05, **p<0.01, ***p<0.001, ****p<0.0001.
**C)** Testis that over-express YAP/YkiGFP in the germline and that are also heterozygous for the null allele wts^x1^.
(TIFF)

S3 Fig Number of GSCs and hub cells in testes expressing GFP or YAP/YkiGFP in the hub. Plot with individual data points showing the number of GSCs (left) and hub cells (right) in testes expressing UAS-GFP (black) or UAS-ykiGFP (purple) by upd-Gal4. The average numbers are:
GSCs control: 7.38±0.29 (n = 13), 5.83±0.70 (n = 6), 6.56±0.24 (n = 9), 5.62±0.31 (n = 13) and 4.50±0.40 (n = 10) at 1, 7, 14, 28 and 50 days, respectively. GSCs Yki: 8.91±0.31 (n = 11), 6.36±0.34 (n = 11), 6.80±0.47 (n = 10), 6.20±0.36 (n = 10), 5.25±0.41 (n = 12) at 1, 7, 14, 28 and 50 days, respectively. Hub control: 8.08±0.38 (n = 13), 7.00±0.26 (n = 6), 6.89±0.61 (n = 9), 6.38±0.40 (n = 13), 5.00±0.26 (n = 10) at 1, 7, 14, 28 and 50 days, respectively. Hub Yki: 9.64±0.56 (n = 11), 7.91±0.56 (n = 11), 8.10±0.48 (n = 10), 7.50±0.31 (n = 10), 5.83±0.27 (n = 12) at 1, 7, 14, 28 and 50 days, respectively.
(TIFF)

S4 Fig Number of GSCs and hub cells in testes expressing GFP or YAP/YkiGFP in CySCs and their progeny. **A)** Testes expressing UAS-GFP (top) or UAS-ykiGFP (bottom), under the somatic driver tjGal4. Tj is expressed in the CySCs and their daughter cyst cells. FasIII and DAPI are red and blue in merge panel, respectively.
**B)** Plot with individual data points showing the number of GSCs (left) and hub cells (right) in testes expressing UAS-GFP (black) or UAS-ykiGFP (purple) by tj-Gal4. The average numbers are: GSCs 1 day UAS-GFP (6.08±0.54, n = 11) or UAS-ykiGFP (6.09±0.48, n = 11); 50 days UAS-GFP (5.00±0.33,n = 10), or UAS-ykiGFP (5.00±0.45, n = 10). Hub cells, 1 day UAS-GFP (7.17±0.52, n = 12) or UAS-ykiGFP (7.27±0.65, n = 11); 50 days UAS-GFP (6.40±0.43,n = 10) or UAS-ykiGFP (6.50±0.45, n = 10).
(TIFF)

S5 Fig Higher levels of E-cadherin in aging testes over-expressing YAP/Yki. **A)** Representative images of E-cadherin in aging testes that express GFP (top) or YAP/Yki (botton) in the germline. DAPI in blue, E-cadh in red or white (right panels), and GFP in green.
**B)** Representative image of PH3 in testes that over-express YAP/Yki in the germline. PH3 in light blue, DAPI (DNA) in dark blue, FasIII in red, and YkiGFP in green.
(TIFF)

S1 Movie Sequence of optical Z-sections of UAS-GFP;nosGal4 testes at the confocal microscope. This movie is representative of the area that was used in this study to quantify GSCs and hub cells. The hub (FasIII) and DNA (DAPI) are red and blue, respectively. The number of GSCs and hub cells were quantified in fixed samples by following DAPI for the nuclei, GFP (as in this movie), or the germline marker Vasa for GSCs and the niche specific marker FasIII for niche cells. GSCs were only counted if they were directly adjacent to the hub structure. For example, this image would result in seven GSCs. Images of planes at various depths representing all GSCs and niche cells were obtained using the Z-stack function of the confocal microscope.
(MOV)

S1 Dataset Raw data. (PDF)
