# Supplementary figures and images for: YAP/Yorkie in the germline modulates the age-related decline of germline stem cells and niche cells
Source: PLoS One. 2019 Apr 3;14(4):e0213327. doi: 10.1371/journal.pone.0213327 (PMC6447158; doi:10.1371/journal.pone.0213327)

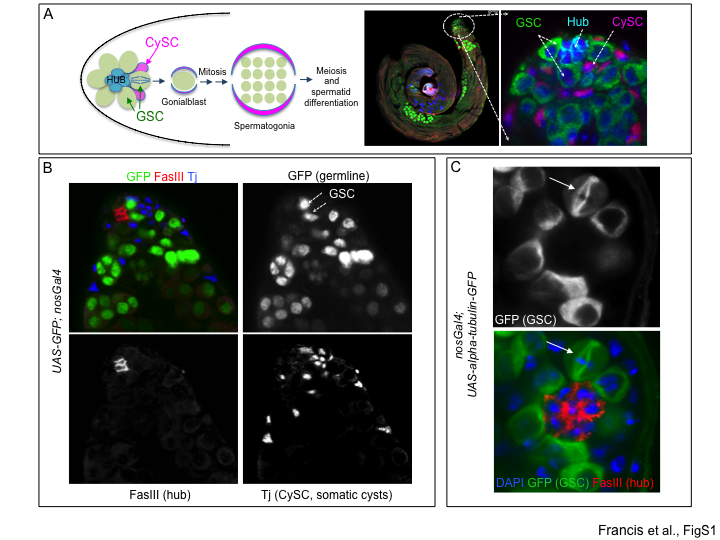

Supplement: S1 Fig — A) Left: scheme of testis. Middle: testis expressing UAS-GFP by the nosGal4 driver. Right: Apical tip: FasIII for hub cells (cyan), Vasa for germline (green), Traffic-jam for somatic lineage (Tj, in magenta), and DNA with DAPI (blue). The Vasa and Tj positive cells in contact with the hub are the GSCs and CySC, respectively. B) UAS-GFP;nosGal4 testes express GFP in all germline cells, including GSCs. The hub is positive for FasIII (red in merge), and the somatic cells (CySC and somatic cyst) for Tj (blue in merge). C) Alpha-tubulin-GFP reveals the orientation of the mitotic spindle in GSCs (arrow). (TIFF) [file pone.0213327.s001.tiff]

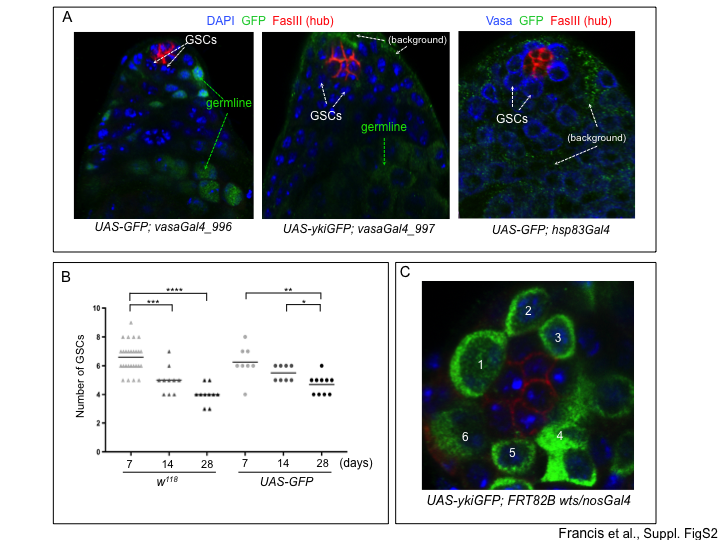

Supplement: S2 Fig — A) Testes expressing GFP by the vasa-Gal4 driver stock DGRC109996 (left), YkiGFP by the vasa-Gal4 driver stock DGRC109997, and GFP by the hsp83-Gal4 driver (right). None of these Gal4 drivers induces expression in the GSCs. B) w118 and UAS-GFP testes show a decrease in the number of GSCs with age. Plot with individual data points showing that the average number of GSCs. This quantification illustrates that in w118 flies, the number of GSCs in 7-days-old testes is 6.59±0.20 (n = 27), with a significant decrease at 14 (5±0.29, n = 10) and 28 days (4±0.21,n = 10). In UAS-GFP flies, the average number of GSCs in 7-days-old testes is 6.25±0.41 (n = 8), with a significant decrease at 14 (5.5±0.18, n = 8) and 28 days (4.7±0.21,n = 10). This age-related reduction in GSC number in w1118 and UAS-GFP 7–28 days-old males was observed by identifying GSCs by their position next to the niche, and by being positive for the germline marker Vasa. *p<0.05, **p<0.01, ***p<0.001, ****p<0.0001. C) Testis that over-express YAP/YkiGFP in the germline and that are also heterozygous for the null allele wtsx1. (TIFF) [file pone.0213327.s002.tiff]

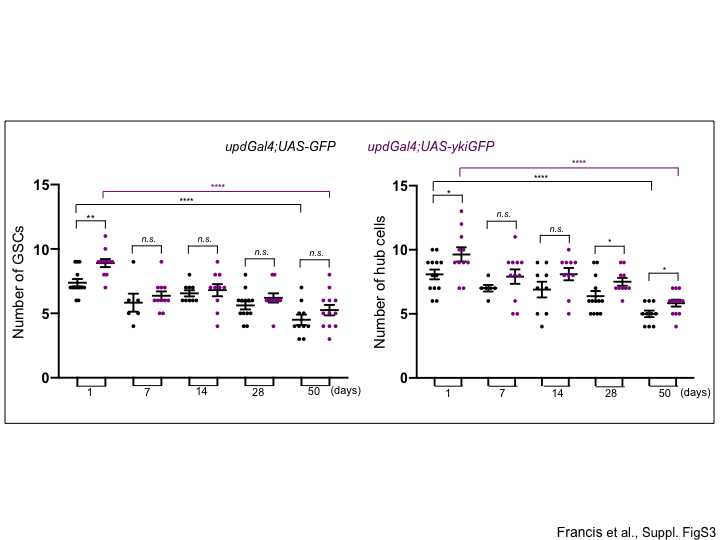

Supplement: S3 Fig — Plot with individual data points showing the number of GSCs (left) and hub cells (right) in testes expressing UAS-GFP (black) or UAS-ykiGFP (purple) by upd-Gal4. The average numbers are: GSCs control: 7.38±0.29 (n = 13), 5.83±0.70 (n = 6), 6.56±0.24 (n = 9), 5.62±0.31 (n = 13) and 4.50±0.40 (n = 10) at 1, 7, 14, 28 and 50 days, respectively. GSCs Yki: 8.91±0.31 (n = 11), 6.36±0.34 (n = 11), 6.80±0.47 (n = 10), 6.20±0.36 (n = 10), 5.25±0.41 (n = 12) at 1, 7, 14, 28 and 50 days, respectively. Hub control: 8.08±0.38 (n = 13), 7.00±0.26 (n = 6), 6.89±0.61 (n = 9), 6.38±0.40 (n = 13), 5.00±0.26 (n = 10) at 1, 7, 14, 28 and 50 days, respectively. Hub Yki: 9.64±0.56 (n = 11), 7.91±0.56 (n = 11), 8.10±0.48 (n = 10), 7.50±0.31 (n = 10), 5.83±0.27 (n = 12) at 1, 7, 14, 28 and 50 days, respectively. (TIFF) [file pone.0213327.s003.tiff]

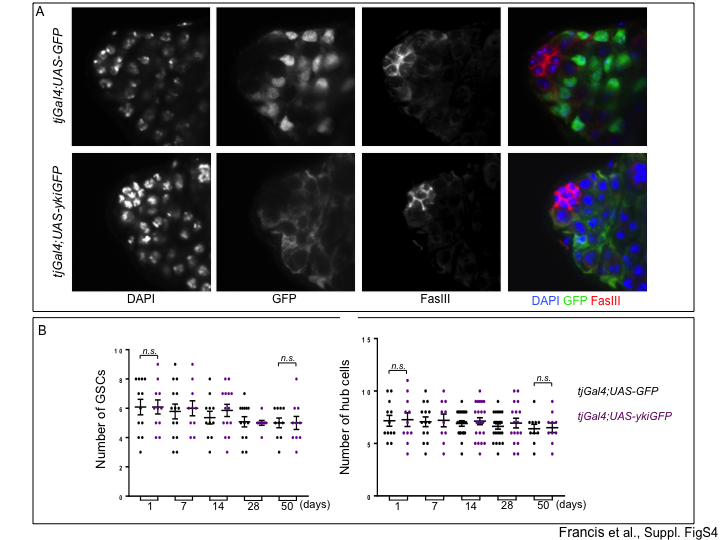

Supplement: S4 Fig — A) Testes expressing UAS-GFP (top) or UAS-ykiGFP (bottom), under the somatic driver tjGal4. Tj is expressed in the CySCs and their daughter cyst cells. FasIII and DAPI are red and blue in merge panel, respectively. B) Plot with individual data points showing the number of GSCs (left) and hub cells (right) in testes expressing UAS-GFP (black) or UAS-ykiGFP (purple) by tj-Gal4. The average numbers are: GSCs 1 day UAS-GFP (6.08±0.54, n = 11) or UAS-ykiGFP (6.09±0.48, n = 11); 50 days UAS-GFP (5.00±0.33,n = 10), or UAS-ykiGFP (5.00±0.45, n = 10). Hub cells, 1 day UAS-GFP (7.17±0.52, n = 12) or UAS-ykiGFP (7.27±0.65, n = 11); 50 days UAS-GFP (6.40±0.43,n = 10) or UAS-ykiGFP (6.50±0.45, n = 10). (TIFF) [file pone.0213327.s004.tiff]

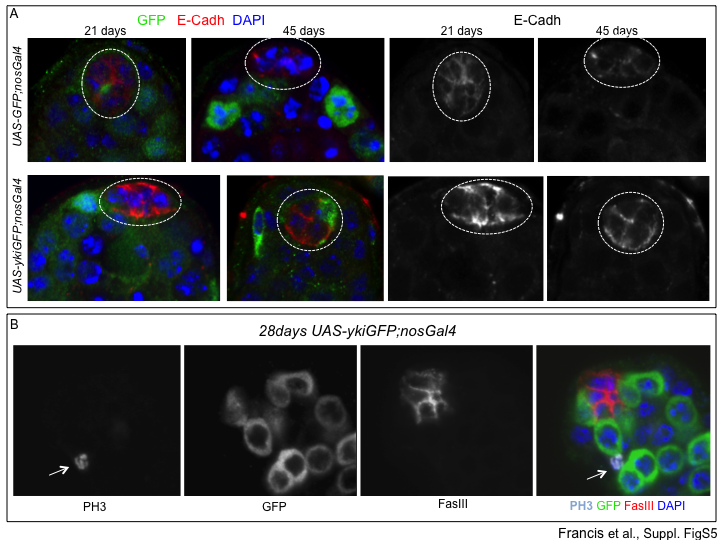

Supplement: S5 Fig — A) Representative images of E-cadherin in aging testes that express GFP (top) or YAP/Yki (botton) in the germline. DAPI in blue, E-cadh in red or white (right panels), and GFP in green. B) Representative image of PH3 in testes that over-express YAP/Yki in the germline. PH3 in light blue, DAPI (DNA) in dark blue, FasIII in red, and YkiGFP in green. (TIFF) [file pone.0213327.s005.tiff]
