# Supplementary material for: YAP/Yorkie in the germline modulates the age-related decline of germline stem cells and niche cells
Source: PLoS One. 2019 Apr 3;14(4):e0213327. doi: 10.1371/journal.pone.0213327 (PMC6447158; doi:10.1371/journal.pone.0213327)
Supplement: S1 Dataset — (PDF) [file pone.0213327.s007.pdf]

1-3 days

	**Hub cells (n)**		**GSCs (n)**	
	nos GFP	nos Yki GFP	nos GFP	nos Yki GFP
	5	5	4	4
	7	8	4	6
	8	7	4	3
	4	8	7	3
	6	7	4	6
	6	3	2	4
	8	8	2	5
	8	12	3	4
	6	5	4	6
	3	10	5	5
	9	8	5	5
	6	9	4	4
	6	5	4	5
	9	8	4	5
	9	6	6	6
	7	6	3	4
	5	6	5	5
	5	7	3	3
	7	7	3	5
	4	9	4	6
	7	5	3	5
	8	6	4	5
	8	5	5	6
	5	6	4	5
	5	8	6	4
	5	3	4	5
	7	5	6	3
	5	5	4	4
	5	4	3	6
	9	7	3	
	3	6		
	6	7		
	6	7		
	5	6		
	5	8		
	6	6		
	7	4		
	7	7		
	7	6		
	6	5		
	6	4		
	8	9		
	8	7		
	5	9		
	5	6		
	7	5		
	8	9		
	3	10		
	7	8		
	5	6		
	7	7		
	6	5		
	6	5		
	8			
MEAN	6.27777778	6.60377358	4.06666667	4.72413793
SD	1.54695888	1.84327909	1.1724814	0.99629857
n	54	53	30	29
SEM	0.21051444	0.25319385	0.21406484	0.185008

7 days

	Hub cells (n)		
	nos GFP	nos Yki GFP	nos Yki RNAi
	6	8	7
	5	6	6
	10	10	5
	5	9	6
	9	9	6
	10	8	4
	6	6	5
	6	5	7
	6	7	7
	7	7	6
	4	8	6
	6	5	6
	5	4	5
	5	6	4
	6	8	
	5	7	
	6	8	
	5	6	
	5	5	
	5	6	
	6	6	
	7	5	
	7	6	
	7	5	
	6		
MEAN	6.2	6.66666667	5.71428571
SD	1.52752523	1.55106322	0.99449032
n	25	24	14
SEM	0.30550505	0.31660945	0.26578872

14 days

	Hub cells (n)		GSCs (n)	
	nos GFP	nos Yki GFP	nos GFP	nos Yki GFP
	5	9	4	6
	5	7	2	5
	5	10	4	4
	8	6	3	4
	7	8	3	7
	5	5	4	5
	6	8	4	3
	8	7	3	5
	6	6	2	5
	6	8	4	4
	7	4	3	4
	4	8	4	4
	8	8	3	3
	7	6	5	4
	5	11	3	9
	5	10		4
	8	9		
	6	8		
	7	7		
	7	6		
	5	6		
	5	7		
	8	8		
	8	11		
	5	7		
	6	9		
	7	8		
	7	7		
	6	10		
	6	7		
	8	12		
	5	9		
	7	7		
	4	7		
	7	7		
	8	6		
	3	6		
	6	7		
	8	6		
		8		
		8		
MEAN	6.25641026	7.65853659	3.4	4.75
SD	1.35176198	1.68240536	0.82807867	1.52752523
n	39	41	15	16
SEM	0.21645515	0.26274757	0.21380899	0.38188131

21 days

	Hub cells (n)			GSCs (n)	
	nos GFP	nos Yki GFP	nos Yki RNAi	nos GFP	nos Yki GFP
	8	10	8	5	7
	7	11	9	4	6
	5	12	5	4	9
	6	6	6	3	4
	7	7	6	5	5
	5	6	3	3	6
	4	4	5	4	5
	7	5	6	5	6
	5	8	7	6	3
	5	6	6	2	5
		7	5		5
		9			6
		11			5
MEAN	5.9	7.84615385	6	4.1	5.53846154
SD	1.28668394	2.54447617	1.61245155	1.197219	1.4500221
n	10	13	11	10	13
SEM	0.40688519	0.70571072	0.48617243	0.37859389	0.40216377

28 days

	Hub cells (n)			GSCs (n)	
	nos GFP	nos Yki GFP	nos Yki RNAi	nos GFP	nos Yki GFP
	5	6	8	2	5
	5	5	5	3	4
	4	6	5	3	8
	7	4	6	4	3
	8	8	4	3	3
	7	7	6	4	3
	8	7	5	3	3
	6	5	6	2	6
	6	6	5	4	6
	5	10	6	3	3
	6	7	3	3	5
	5	6	8	2	4
	8	6	8	3	8
	8	8	6	3	3
	6	6	5	2	5
	6	8	6	2	7
	8	7	5	2	9
	6	9	4	5	6
	5	7	5	5	5
	6	5	7	7	8
	6	7	6	5	9
	6	7		4	8
	10	7		5	8
	4	13		3	9
	6	6		5	8
	6	7		5	5
	8	8		3	7
	5	8		6	10
	9	9		3	7
	6	8		4	9
	8	8		4	6
	8	10		5	7
	7	8		6	10
	9	9		3	7
	6	9		4	7
	8	7		5	5
	12	8		4	8
	8	7		5	5
	7	11		4	8
	6	9		3	8
	6	10		5	5
	6	11		6	8
	8	8		4	8
	8	9		3	6
	6	9		4	5
	5	10		5	10
	7	11		4	4
	7	9		3	7
	9	11		4	9
	9	9		5	5
	6	10		6	10
	9	9		4	6
	8	12		3	8
	8	10		6	7
	7	12		6	8
	6	10		5	7
	9	9		5	4
	6	13		7	6
	8	10		5	5
	7	7		4	4
	8	9		4	5
	9	9		5	5
	6	9		4	6
	5	8			4
	5	9			5
	6	8			4
	5	10			4
	6	8			
	5	9			
	4	10			
	5	9			
	6	7			
	7	8			
	5	9			
		8			
		11			
		11			
		9			
		8			
		9			
		9			
		11			
		9			
		6			
		7			
		7			
		6			
		7			
		6			
		7			
		5			
		8			
MEAN	6.71621622	8.30434783	5.66666667	4.0952381	6.26865672
SD	1.54848817	1.83817355	1.31656118	1.25356634	2.00440492
n	74	92	21	63	67
SEM	0.18000809	0.19164284	0.2872972	0.15793451	0.24487704

35 days

	Hub cells (n)			GSCs (n)	
	nos GFP	nos Yki GFP	nos Yki RNAi	nos GFP	nos Yki GFP
	8	9	7	7	5
	6	8	7	4	5
	5	6	6	6	5
	5	6	6	4	4
	6	7	6	4	5
	6	6	5	3	4
	5	6	6	6	4
	6	5	6	5	6
	5	7	5	4	4
	4	9	7	3	6
MEAN	5.6	6.9	6.1	4.6	4.8
SD	1.0749677	1.37032032	0.73786479	1.34989712	0.78881064
n	10	10	10	10	10
SEM	0.33993463	0.43333333	0.23333333	0.42687495	0.24944383

42-54 days

	Hub cells (n)			GSCs (n)	
	nos GFP	nos Yki GFP	nos Yki RNAi	nos GFP	nos Yki GFP
	6	7	9	4	6
	4	11	6	5	3
	5	7	5	3	5
	7	6	7	3	3
	7	8	6	2	3
	5	8	5	2	4
	7	9	5	3	6
	5	9	3	3	4
	6	9	5	3	5
	6	10	6	3	4
	4	9	4	4	5
	3	8	6	2	4
	4	9	5	3	5
	4	6	5	3	6
	6	7	5	6	4
	6	3	6	4	4
	4	8	6	3	6
	6	8	5	7	7
	6	5	6	3	7
	4	9	3	3	5
	7	7	4	5	4
	5	7	5	3	3
	4	6	5	3	4
	5	5		3	4
	3	4		3	4
	4	3		3	3
	5	7		4	6
	6	6		4	6
	6			3	
MEAN	5.17241379	7.17857143	5.30434783	3.44827586	4.64285714
SD	1.19728757	1.98239607	1.25895998	1.12078438	1.22366437
n	29	28	23	29	28
SEM	0.22233072	0.37463764	0.2625113	0.20812444	0.23125083

nlsGFP 14 days EdU

Hub cells	GSCs	EdU labelled	% Incorporation	GFP	EdU
7	4	3	75		
8	4	4	100		
5	3	0	0		
7	4	0	0		
8	5	0	0		
11	5	5	100		
8	4	0	0		
7	4	0	0	1	
5	2	2	100		
6	4	4	100		
6	3	3	100		
7	4	4	100	1	
7	3	0	0		
6	3	3	100	1	
8	5	0	0		
6	3	0	0	1	
7	4	0	0		
8	4	0	0		
7	4	4	100		
8	5	0	0		
11	6	6	100		
6	3	3	100		
7	3	3	100		
7	5	0	0		
6	3	3	100	1	
6	4	0	0	1	

No. of EdU labelled sampl	13 of 26		max		min	
50%	% incorporation			26.92		23.08

nlsGFP 28 days EdU

Hub cells	GSCs	EdU labelled	% Incorporation	GFP	EdU
6	3	0	0		
7	5	5	100		
8	4	0	0		
6	4	2	50		
7	3	0	0	1	
7	5	0	0	1	
8	4	0	0		
7	5	0	0		
8	4	0	0		
7	4	0	0		
6	4	0	0		
7	3	0	0	1	
6	3	0	0		
6	3	0	0		
7	3	0	0	1	
5	3	3	100		
8	4	2	50		
7	4	1	25		
7	3	0	0		
6	3	0	0		
8	4	3	75		
5	3	0	0		
5	2	2	100		
9	4	0	0	1	
8	5	0	0	1	

No. of EdU labelled Sampl	7 of 25		max		min	
28	% incorporation			40		24

YkiGFP 14 days EdU

Hub cells	GSCs	EdU labelled	%	Incorporation GFP	EdU
7	7	6	85.7		
9	8	0	0.0		
7	4	4	100.0	1	
10	5	4	80.0	1	46
9	8	0	0.0		
9	5	4	80.0		
8	6	4	66.7	1	
10	5	0	0.0		
11	8	7	87.5		
9	6	6	100.0		
10	1	1	100.0		
7	8	8	100.0		
9	6	5	83.3		
7	5	5	100.0		
7	5	5	100.0		
6	4	0	0.0	1	
8	5	0	0.0		
8	4	4	100.0		
7	7	6	85.7		
7	4	4	100.0		
10	6	6	100.0	2	
9	7	7	100.0		
10	4	4	100.0		
8	5	5	100.0	1	
10	4	4	100.0	1	49
8	5	5	100.0		7
8	4	4	100.0		
9	5	0	0.0	x	
				min	max
No. of EdU labelled sampl		22 of 28		25	28.57
78.57	% incorporation				

YkiGFP 28 days EdU

Hub cells	GSCs	EdU labelled	%	Incorporation GFP	EdU
9	8	7	87.5		2
9	7	0	0.0		6
8	9	0	0.0		
7	5	3	60.0		10
9	6	4	66.7		
10	8	5	62.5	1	18
8	5	0	0.0		
10	6	0	0.0		21
6	3	2	66.7		25
9	9	0	0.0	1	28
9	6	0	0.0	1	31
8	7	1	14.3		34
7	5	2	40.0		
7	4	4	100.0		37
7	6	0	0.0		
8	6	3	50.0		
7	5	2	40.0		
8	5	2	40.0		7
9	4	0	0.0	1	9
8	7	0	0.0		
8	5	4	80.0	1	13
8	4	1	25.0	1	16
8	4	0	0.0		
6	3	3	100.0		
8	5	0	0.0	2	22
8	6	0	0.0		
No. of EdU labelled sampl		14 of 26		min	max
53.85	% incorporation			26.92	38.46

1 day

	Hub cells (n)		GSCs (n)	
	nos GFP wts	nos Yki wts	nos GFP wts	nos Yki wts
	8	12	7	9
	9	10	9	8
	8	8	9	6
	8	10	3	9
	8	10	5	8
	9	11	7	9
	10	11	7	8
	10	8	6	5
	9	13	6	9
	8	12	7	8
	10		7	
	10		8	
	6		5	
	7		5	
MEAN	8.57142857	10.5	6.5	7.9
SD	1.22249969	1.64991582	1.65250393	1.37032032
n	14	10	14	10
SEM	0.32672679	0.52174919	0.44165025	0.43333333

7 days

	**Hub cells (n)**		**GSCs (n)**	
	nos GFP wts	nos Yki wts	nos GFP wts	nos Yki wts
	7	10	7	6
	7	8	5	9
	8	9	6	7
	7	9	6	5
	7	9	8	7
	6	9	5	5
	6	8	6	5
	6	10	4	6
	5	9	4	8
	5	12	5	8
	6	10	3	7
	6	9	5	4
	7	9	4	7
	6	9	5	6
	5		4	
	7		5	
	5		3	
	6		4	
	5		3	
MEAN	6.15789474	9.28571429	4.84210526	6.42857143
SD	0.89834155	0.99449032	1.34425353	1.39858641
n	19	14	19	14
SEM	0.20609369	0.26578872	0.30839291	0.37378794

28 days

	Hub cells (n)		GSCs (n)	
	nos GFP wts	nos Yki wts	nos GFP wts	nos Yki wts
	9	9	6	4
	10	8	6	6
	8	8	5	5
	8	9	6	3
	10	8	5	6
	9	9	5	7
	9	9	5	7
	7	9	4	6
	8	10	5	7
	9	10	5	8
	9	8	3	4
	6	9	3	6
	8	10	4	8
	10	12	5	6
	9	9	3	5
	10	10	5	5
	8	12	7	6
	9		5	
MEAN	8.66666667	9.35294118	4.83333333	5.82352941
SD	1.08465229	1.22173936	1.09812675	1.38000426
n	18	17	18	17
SEM	0.255655	0.29631532	0.25883096	0.3347002

1 day

	Hub cells (n)		GSCs (n)	
	upd GFP	upd Yki GFP	upd GFP	upd Yki GFP
	6	12	9	9
	10	7	9	11
	9	10	6	9
	8	10	8	9
	6	9	7	8
	8	11	7	9
	7	9	7	7
	9	9	7	8
	9	9	7	9
	7	7	9	9
	10	13	7	10
	9		7	
	7		6	
MEAN	8.07692308	9.63636364	7.38461538	8.90909091
SD	1.38212026	1.85864075	1.04390785	1.04446594
n	13	11	13	11
SEM	0.38333119	0.56040127	0.28952794	0.31491833

7 days

	**Hub cells (n)**		**GSCs (n)**	
	upd GFP	upd Yki GFP	upd GFP	upd Yki GFP
	7	8	9	5
	8	9	6	7
	7	8	5	6
	7	9	5	7
	7	11	6	9
	6	8	4	6
		6		6
		9		7
		9		6
		5		6
		5		5
MEAN	7	7.90909091	5.83333333	6.36363636
SD	0.63245553	1.86839747	1.72240142	1.12006493
n	6	11	6	11
SEM	0.25819889	0.56334303	0.70316744	0.33771228

14 days

	**Hub cells (n)**		**GSCs (n)**	
	upd GFP	upd Yki GFP	upd GFP	upd Yki GFP
	8	9	7	4
	9	8	8	8
	7	6	7	5
	5	8	7	9
	9	9	6	8
	7	8	6	7
		5		7
	8	9	6	6
	5	10	6	7
	4	9	6	7
MEAN	6.88888889	8.1	6.55555556	6.8
SD	1.83333333	1.52388393	0.72648316	1.47572957
n	9	10	9	10
SEM	0.61111111	0.48189441	0.24216105	0.46666667

28 days

	Hub cells (n)		GSCs (n)	
	upd GFP	upd Yki GFP	upd GFP	upd Yki GFP
	9	7	6	4
	9	9	8	8
	5	8	7	6
	7	7	6	6
	8	9	6	6
	6	7	6	6
	6	8	5	6
	5	7	5	8
	5	6	4	6
	6	7	5	6
	6		6	
	6		5	
	5		4	
MEAN	6.38461538	7.5	5.61538462	6.2
SD	1.44559455	0.97182532	1.12089708	1.13529242
n	13	10	13	10
SEM	0.40093579	0.30731815	0.31088091	0.35901099

50 days

	**Hub cells (n)**		**GSCs (n)**	
	upd GFP	upd Yki GFP	upd GFP	upd Yki GFP
	6	7	7	7
	4	6	5	5
	6	6	4	6
	4	4	3	4
	6	5	4	8
	5	6	5	4
	4	7	3	6
	5	5	4	4
	5	6	6	5
	5	5	4	3
		7		6
		6		5
MEAN	5	5.83333333	4.5	5.25
SD	0.81649658	0.93743687	1.26929552	1.42222617
n	10	12	10	12
SEM	0.25819889	0.27061471	0.40138649	0.41056133

1 day

	Hub cells (n)		GSCs (n)	
	TJ GFP	TJ Yki GFP	TJ GFP	TJ Yki GFP
	9	11	8	9
	10	7	7	8
	6	10	4	7
	6	6	8	4
	7	8	5	6
	5	6	7	7
	8	9	6	6
	6	6	4	6
	5	8	3	5
	10	5	8	4
	6	4	8	5
	8		5	
MEAN	7.16666667	7.27272727	6.08333333	6.09090909
SD	1.80067327	2.14899554	1.88775961	1.57826141
n	12	11	12	11
SEM	0.5198096	0.64794653	0.54494926	0.47586372

7 days

	Hub cells (n)		GSCs (n)	
	TJ GFP	TJ Yki GFP	TJ GFP	TJ Yki GFP
	8	8	4	8
	6	10	7	9
	4	4	3	5
	6	7	5	4
	7	6	6	6
	8	9	8	6
	5	5	9	7
	10	6	7	5
	7	8	3	4
	9	9	8	6
	5		5	
	8		6	
	7		4	
	9		6	
MEAN	7.07142857	7.2	5.78571429	6
SD	1.73046395	1.93218357	1.8883681	1.63299316
n	14	10	14	10
SEM	0.46248595	0.61101009	0.5046876	0.51639778

14 days

	Hub cells (n)		GSCs (n)	
	TJ GFP	TJ Yki GFP	TJ GFP	TJ Yki GFP
	7	7	5	7
	8	5	6	7
	6	9	5	8
	7	10	7	5
	4	7	6	7
	6	7	5	5
	8	7	6	6
	5	8	3	4
	7	5	4	7
	8	8	8	3
	8	6	4	5
	8	8		6
	9	4		8
	8	5		4
	7	9		
	6	7		
	6	6		
	8	8		
	6	9		
	6	7		
	7	6		
		5		
		9		
		9		
MEAN	6.9047619	7.04761905	5.36363636	5.85714286
SD	1.2208506	1.62353613	1.43336857	1.56190923
n	21	24	11	14
SEM	0.26641144	0.33140292	0.43217688	0.4174378

28 days

	Hub cells (n)		GSCs (n)	
	TJ GFP	TJ Yki GFP	TJ GFP	TJ Yki GFP
	8	4	4	5
	6	10	6	5
	7	5	7	5
	6	7	6	5
	7	8	6	6
	7	7	5	5
	5	6	3	4
	8	8	5	5
	7	8	6	5
	5	6	5	
	7	9	6	
	6	5	3	
	8	10	4	
	5	9		
	4	6		
	5	4		
	6	6		
	8			
	7			
	9			
	6			
	7			
	7			
	8			
MEAN	6.625	6.94117647	5.07692308	5
SD	1.24455335	1.91932893	1.25575598	0.5
n	24	17	13	9
SEM	0.25404339	0.46550564	0.34828404	0.16666667

50 days

	Hub cells (n)		GSCs (n)	
	TJ GFP	TJ Yki GFP	TJ GFP	TJ Yki GFP
	5	8	6	5
	6	9	3	4
	4	4	4	8
	9	5	4	4
	6	7	6	3
	7	6	5	5
	7	6	6	4
	7	7	5	5
	6	7	6	6
	7	6	5	6
MEAN	6.4	6.5	5	5
SD	1.34989712	1.43372088	1.05409255	1.41421356
n	10	10	10	10
SEM	0.42687495	0.45338235	0.33333333	0.4472136
